# Topical medium-length PDRN enhances dermal extracellular matrix repair in photodamaged skin via PI3K–Akt/TGF-β–regulated pathways

**DOI:** 10.1371/journal.pone.0350905

**Published:** 2026-07-10

**Authors:** Rui Ye, Qianqian Wang, Le Du, Li Li, Fan Hu

**Affiliations:** 1 Department of Dermatology & Venereology, Center of Cosmetic Safety and Efficacy Evaluation and NMPA Key Laboratory for Human Evaluation and Big Data of Cosmetics, West China Hospital, Sichuan University, Chengdu, China; 2 UNISKIN Research Institute on Skin Aging, Inertia Shanghai Biotechnology Co., Ltd., Shanghai, China; 3 DermaHealth Shanghai Biotechnology Co., Ltd., Shanghai, China; 4 Department of Dermatology, Huashan Hospital, Fudan University, Shanghai, China; POSTECH - Pohang University of Science and Technology, KOREA, REPUBLIC OF

## Abstract

Chronic ultraviolet (UV) exposure disrupts dermal fibroblast function and extracellular matrix (ECM) homeostasis, driving the structural features of photoaged skin. Polydeoxyribonucleotide (PDRN) has shown regenerative effects in injectable applications, but the mechanism and delivery of topical PDRN remain insufficiently defined. This study investigated the mechanism, skin delivery, and efficacy of a medium-length PDRN preparation (PDRN-850K) in photodamaged skin. Pathway activation was examined in basal human dermal fibroblasts using western blotting, gene expression analysis, and pharmacological inhibition. Skin delivery was assessed by confocal Raman spectroscopy in a reconstructed epidermal model and by complementary ex vivo and in vivo penetration studies. Tissue-level effects were assessed in a UV-irradiated human ex vivo skin model. Clinical efficacy was evaluated in a randomized, double-blind, split-face study comparing 0.1% PDRN-850K eye cream with 0.1% retinol over 28 days. PDRN-850K activated PI3K–Akt, TGF-β/Smad, and autophagy-related signaling in basal fibroblasts, and inhibition of these pathways reduced ECM gene induction. Raman analysis demonstrated time-dependent distribution of PDRN-associated signal into viable epidermal regions, with additional support from FITC-labeled porcine skin and pilot in vivo human Raman studies. In UV-irradiated ex vivo skin, PDRN-850K increased viable epidermal thickness and upregulated multiple collagens, elastic fiber–associated proteins, and YAP. Clinically, PDRN-850K achieved approximately two-fold greater improvements in periocular wrinkles, dermal thickness, density, and eye bag parameters compared with retinol, with good tolerability. These findings support PDRN-850K as a topical pro-repair approach for photodamaged skin.

## Introduction

Skin photoaging is driven largely by chronic ultraviolet (UV) exposure, which impairs dermal fibroblast function and disrupts extracellular matrix (ECM) homeostasis. These changes contribute to collagen loss, elastin disorganization, wrinkle formation, and reduced skin elasticity [1–4].

Polydeoxyribonucleotide (PDRN) is a DNA-derived polymeric biomaterial with reported regenerative, pro-survival, and tissue-repair effects [5–8]. Most studies and clinical applications of PDRN have focused on injectable use. In contrast, the biological activity and delivery behavior of topically applied PDRN remain insufficiently characterized, particularly in photodamaged skin. Because PDRN is a high-molecular-weight nucleic acid polymer, whether it can reach viable skin layers after topical application remains an important translational question.

At the cellular level, ECM production and remodeling are governed by coordinated signaling networks that regulate fibroblast survival, biosynthetic capacity, and stress adaptation [9,10]. The phosphoinositide 3-kinase (PI3K)–Akt pathway supports fibroblast viability and anabolic activity [11,12], while transforming growth factor-β (TGF-β) signaling through Smad transcription factors represents a canonical driver of collagen synthesis and matrix organization [13,14]. In parallel, autophagy serves as a critical homeostatic mechanism that maintains proteostasis and cellular competence under stress conditions relevant to photoaging [15,16]. In our previous transcriptomic study, treatment with a medium-length PDRN preparation was associated with enrichment of these pathways together with restoration of ECM-related gene expression in UV-damaged fibroblast models [17]. However, direct pathway-level evidence and functional validation have remained limited.

In the present study, we focused on the same medium-length PDRN preparation with an average molecular weight not exceeding 850 kDa (hereafter referred to as PDRN-850K), which was previously shown to promote dermal ECM restoration under photodamage-relevant conditions. Building on these findings, we investigated its effects on PI3K-Akt, TGF-β/Smad, and autophagy-related signaling in human dermal fibroblasts; evaluated its skin delivery using reconstructed epidermis, ex vivo porcine skin, and in vivo human Raman approaches; and assessed its tissue-level and clinical effects in UV-damaged ex vivo skin and a randomized split-face clinical study. Our aim was to clarify whether topical PDRN-850K can support dermal ECM repair in photodamaged skin and to provide mechanistic, penetration, and translational evidence for its activity.

## Materials and methods

### Materials

PDRN-850K used in this study was provided by Ruijiming (Shandong) Biotechnology Co., Ltd. The material is a high-purity (≥96%) polydeoxyribonucleotide extracted from salmon sperm, with an average fragment length of approximately 1200 base pairs and an average molecular weight not exceeding 850 kDa. PDRN-850K was supplied as a lyophilized powder and freshly reconstituted in sterile water or appropriate culture medium immediately prior to use in all experiments. Retinol (ROL) was kindly provided by DSM-Firmenich.

### Cell culture and treatments

Human dermal fibroblasts (HDFs) were cultured in Dulbecco’s Modified Eagle Medium (DMEM; Cellmax, China) supplemented with 10% fetal bovine serum and 1% penicillin–streptomycin, and maintained at 37 °C in a humidified atmosphere with 5% CO2. For protein expression analysis, HDFs were treated with PDRN-850K for 6 h prior to harvesting. For pathway inhibition experiments, cells were pre-incubated for 1 h with LY294002 (20 μM; PI3K inhibitor), SB-431542 (10 μM; TGF-β receptor inhibitor), or chloroquine (40 μM; autophagy inhibitor), followed by co-treatment with PDRN-850K for 48 h before downstream analyses.

### Western blot

Cells were lysed in RIPA buffer containing protease and phosphatase inhibitors, and protein concentrations were determined using a BCA assay. Equal amounts of protein were separated by SDS–PAGE (10% or 15%, depending on target molecular weight) and transferred onto PVDF membranes. Membranes were blocked with 5% bovine serum albumin and incubated overnight at 4 °C with primary antibodies against β-actin, AKT, phospho-AKT, PI3K p85, phospho-PI3K p85, Smad2/3, phospho-SMAD2, phospho-SMAD3, SQSTM1/p62, TGF-β, and LC3A/B. After incubation with HRP-conjugated secondary antibodies, immunoreactive bands were visualized using enhanced chemiluminescence and quantified using ImageJ software.

### Quantitative real-time PCR

Total RNA was extracted using TRIzon reagent and quantified fluorometrically, followed by reverse transcription into cDNA. Quantitative PCR was performed using SYBR Green chemistry on a qTOWER384 real-time PCR system. All reactions were conducted in duplicate. Relative gene expression was calculated using the 2 ⁻ ΔΔCt method, with primer sequences listed in Table 1.

**Table 1:** Primer sequences.

Name	Primer	5’-3’
*COL1A1*	F	GTGCGATGACGTGATCTGTGA
	R	CGGTGGTTTCTTGGTCGGT
*COL3A1*	F	TTGAAGGAGGATGTTCCCATCT
	R	ACAGACACATATTTGGCATGGTT
*ELN*	F	GCAGGAGTTAAGCCCAAGG
	R	TGTAGGGCAGTCCATAGCCA
*MMP1*	F	GGGGCTTTGATGTACCCTAGC
	R	TGTCACACGCTTTTGGGGTTT
*BCL3*	F	GAACACCGAGTGCCAAGAAACC
	R	GCTAAGGCTGTTGTTTTCCACGG
*SMAD3*	F	TGAGGCTGTCTACCAGTTGACC
	R	GTGAGGACCTTGTCAAGCCACT
*TGFBI*	F	GGACATGCTCACTATCAACGGG
	R	CTGTGGACACATCAGACTCTGC
*GAPDH*	F	GGAGCGAGATCCCTCCAAAAT
	R	GGCTGTTGTCATACTTCTCATGG

### UV-irradiated human ex vivo skin model

Human ex vivo full-thickness skin explants were cultured using a commercial human skin culture system. Samples were placed in culture inserts in 6-well plates containing 3.7 mL of skin culture medium per well and maintained at 37 °C in a humidified atmosphere with 5% CO_2_. Culture medium was replaced daily. After a 2-day equilibration period, skin explants were randomly assigned to experimental groups (Table 2).

**Table 2:** Test groups for UV-irradiated human ex vivo skin model.

Group name	Treatment
Blank control (BC)	No UV irradiation, cultured in fresh medium only
Negative control (NC)	UV-irradiated (30 J/cm^2^ UVA + 50mJ/cm^2^ UVB), cultured in fresh medium
Positive control (PC)	UV-irradiated, treated with vitamin C and vitamin E (VC + VE; 100 μg/mL and 7 μg/mL, respectively)
PDRN-850K eye cream (PDRN-850K)	UV-irradiated, treated with 0.1% PDRN-850K eye cream formulation

The test product was a leave-on water-in-silicone eye cream formulated for periocular application, containing 0.1% (w/w) PDRN-850K as the sole bioactive ingredient. All other components consisted of standard cosmetic excipients selected for formulation stability and skin compatibility.

### UVA/UVB irradiation and treatment protocol

Following equilibration, skin explants were exposed to combined UVA and UVB irradiation under standardized, calibrated conditions, except for the blank control (BC) group, which was not irradiated. Immediately after irradiation, treatments were applied according to group assignment: the negative control (NC) group received fresh culture medium only; the positive control (PC) group received culture medium supplemented with vitamin C and vitamin E; and the sample group received topical application of the test formulation. After irradiation and treatment, skin explants were incubated for 24 h at 37 °C with 5% CO₂. This irradiation–treatment cycle was repeated four times. Following completion of irradiation cycles, the BC and NC groups continued to receive daily medium replacement, while the PC and sample groups received daily treatment for an additional 3 days.

### Histological and immunostaining analyses

For morphological assessment, skin samples were fixed in 4% paraformaldehyde, embedded, sectioned, and stained with hematoxylin and eosin (H&E). Tissue morphology was examined by light microscopy. For protein expression analysis, paraformaldehyde-fixed sections were processed for immunofluorescence or immunohistochemistry using antibodies against Collagen I, III, IV, V, VI, VII, XV, XVII, and XVIII, elastin, fibrillin-1, and Yes-associated protein (YAP). Stained sections were imaged by fluorescence or bright-field microscopy, and signal intensities were quantified by image analysis.

### Quantification and statistical analysis

Protein expression was quantified by integrated optical density (IOD) and normalized to the blank control (BC), set to 1.0. Relative changes were calculated as:


$$\begin{array}{c} \text{Relative change }(\%)=\frac{\text{Sample group}-\text{Negative control}}{\text{Negative control}}\times \text{1}\text{0}\text{0}\% \end{array}$$

Data are presented as mean ± SD. Statistical methods are described in the Statistical analysis section.

### 3D skin penetration analysis by confocal Raman spectroscopy

The skin penetration behavior of PDRN-850K was evaluated using a three-dimensional human epidermal model (EpiKutis®, Guangdong Biocell, China). PDRN-850K was prepared as an aqueous solution (250 ppm) and topically applied to the epidermal surface at a fixed volume of 10 µL per insert. PDRN-850K solution was designed as a simplified mechanistic penetration model, not as the sole formulation-translational penetration assay. To assess the involvement of cellular uptake processes, parallel groups were co-treated with the endocytosis inhibitor cytochalasin D (1, 5, or 10 µM; MedChemExpress, USA), administered via the basal culture medium. Untreated models served as controls.

Skin models were incubated at 37 °C with 5% CO2 for 2 h, 6 h, or 24 h, then cryo-fixed and sectioned. Confocal Raman measurements were performed using a three-dimensional confocal Raman microscope (InVia Qontor, Renishaw). Depth-resolved Raman spectra were collected across the epidermal thickness, and PDRN distribution was assessed using the characteristic nucleic acid Raman peak at 1081 cm ⁻ ¹. Time points were selected to capture baseline and a practical early-to-late kinetic profile of topical PDRN-850K delivery in the reconstructed epidermis. An untreated baseline (0 h) was used as the reference for background signal, while 2 h, 6 h, and 24 h were chosen to represent early, intermediate, and late post-application intervals, respectively.

Raman data were processed using WITec Project software with baseline correction and spectral smoothing. Relative penetration amount (arbitrary units) and maximal penetration depth were quantified from integrated peak intensity profiles. Data are presented as mean ± SD, and statistical methods are described in the Statistical analysis section.

### Ex vivo penetration of FITC-labeled PDRN in porcine skin

PDRN-850K was labeled with fluorescein isothiocyanate (FITC) by incubating FITC with PDRN-850K under mildly alkaline conditions at 4 °C overnight. The reaction mixture was dialyzed using a 3500 Da molecular weight cutoff membrane to remove unreacted FITC, and the labeled product was lyophilized.

For the penetration study, FITC-labeled PDRN-850K was reconstituted in deionized water to a final PDRN-850K concentration of 0.1% (1000 ppm). Fresh porcine dorsal skin was mounted in Franz diffusion cells with the stratum corneum facing the donor chamber. The receptor chamber was filled with PBS (pH 7.4) and maintained at 32 ± 1 °C under continuous stirring. After topical application of the FITC-labeled PDRN-850K solution, skin samples were collected after 4, 8, or 12 h, rinsed with PBS, fixed in 4% paraformaldehyde, embedded in OCT, and cryosectioned perpendicular to the skin surface. Fluorescence distribution across skin layers was then examined by confocal laser scanning microscopy.

### In vivo skin penetration analysis by confocal Raman spectroscopy

An exploratory in vivo Raman study was performed using the 0.1% PDRN-850K water-in-silicone eye cream (same formulation as used in the ex vivo study). One healthy volunteer was tested on a 2 × 2 cm area of the volar forearm. The test site was cleaned with water, and the subject acclimated for 30 min in a controlled environment (22 ± 2 °C, 50% ± 10% relative humidity) before product application. Raman measurements were obtained at baseline and at 4, 8, and 24 h after application.

Depth-resolved Raman measurements were performed using a LabRAM Odyssey confocal Raman microscope (HORIBA). Acquisition was conducted in the X–Z direction with point-by-point scanning, using a laser power of 2.68 mW, an integration time of 0.5 s per point, a depth step of 5 μm, and a scan area of 20 × 120 μm.

Raman spectra were preprocessed by cosmic-ray removal, smoothing, background subtraction, baseline correction, and normalization. Baseline intrinsic skin spectra were first characterized, with major endogenous peaks observed at 943, 1275, 1455, 1655, 2846, 2883, 2934, and 3226 cm ⁻ ¹. Product-associated penetration was then tracked using the characteristic Raman spectral profile of PDRN-850K in the eye cream, including peaks at 507, 785, 1106, 1247, 1378, 1479, 1574, 1672, 1901, 2965, and 3175 cm ⁻ ¹, which were distinguishable from the intrinsic skin spectrum. Relative penetration was calculated as the normalized change in product-associated Raman features after application and expressed as a percentage. Because this was a single-subject pilot study, the results were interpreted descriptively.

### Clinical evaluation of 0.1% PDRN-850K eye cream versus 0.1% Retinol eye cream

This was a prospective, randomized, double-blind, split-face, self-controlled clinical study conducted in healthy Chinese female volunteers. The study protocol was reviewed and approved by the Ethics Committee of Hangzhou Sinotek Quality Technology Service Co., Ltd. (Approval No.: (2025) EC-Rev. (19); Project No.: 250924; approved 25 September 2025). The study was conducted in accordance with the Declaration of Helsinki guidelines. Written informed consent was obtained from all participants prior to enrollment. The start and end of the recruitment period for this study was from 28 September 2025–29 September 2025.

Thirty-one healthy Chinese female volunteers aged 35–55 years (mean age 47.5 ± 5.7 years) with self-reported sensitive skin were enrolled. Sensitive skin was defined by Huaxi Questionnaire scores of 18–33. All participants presented bilateral periocular fine lines or wrinkles and eye bags, with crow’s feet and infraorbital wrinkle grades > 2 and eye bag grades > 1 according to the *Skin Age Atlas: Asian Type, Volume 2*. Key exclusion criteria included pregnancy or lactation; systemic or severe dermatological disease; known allergy to retinol or PDRN; and use of periocular products containing retinoids or nucleic acid–based actives within 3 months prior to enrollment.

Participants applied an eye cream containing 0.1% PDRN-850K (same formulation as used in the ex vivo study) to one periocular side and an eye cream with an identical base containing 0.1% retinol to the contralateral side. Products were applied twice daily for 4 weeks following facial cleansing, using a standardized amount and identical application procedures. Daytime sunscreen use was required throughout the study.

Clinical evaluations were performed at baseline (W0), day 14 (D14), and day 28 (D28). Before measurements, participants acclimated for ≥20 min in a controlled environment (21 ± 1 °C; 50 ± 10% relative humidity). Periocular wrinkles were assessed using VISIA 7 (Canfield, USA) with quantitative analysis of wrinkle number and area (Image-Pro Plus). Tear trough and eye bag volume were evaluated using Antera 3D (Miravex, Ireland). Skin firmness (F4) and elasticity (R2) were measured with a Cutometer MPA580 (2-mm probe), and dermal thickness and density were assessed using Ultrascan UC22 (Courage + Khazaka, Germany).

Data are presented as mean ± standard error (SE). Statistical methods are described in the Statistical analysis section.

### Statistical analysis

Statistical analyses were performed using GraphPad Prism and SPSS 26.0 software. Data are presented as mean ± SD unless otherwise stated. Normality was assessed using the Shapiro–Wilk test. For datasets involving more than two groups under a single experimental factor, statistical significance was evaluated by one-way ANOVA followed by appropriate post hoc multiple-comparison tests. For datasets involving two experimental factors, such as treatment and time, two-way ANOVA with appropriate post hoc multiple-comparison tests was used. For the split-face clinical study, direct paired comparisons between two conditions were performed using paired t-tests for normally distributed data and Wilcoxon signed-rank tests for non-normal data. All statistical tests were two-sided, and p < 0.05 was considered statistically significant.

## Results

### PDRN-850K activates PI3K–Akt, TGF-β/Smad, and autophagy-related signaling in basal human dermal fibroblasts

**Fig 1:**
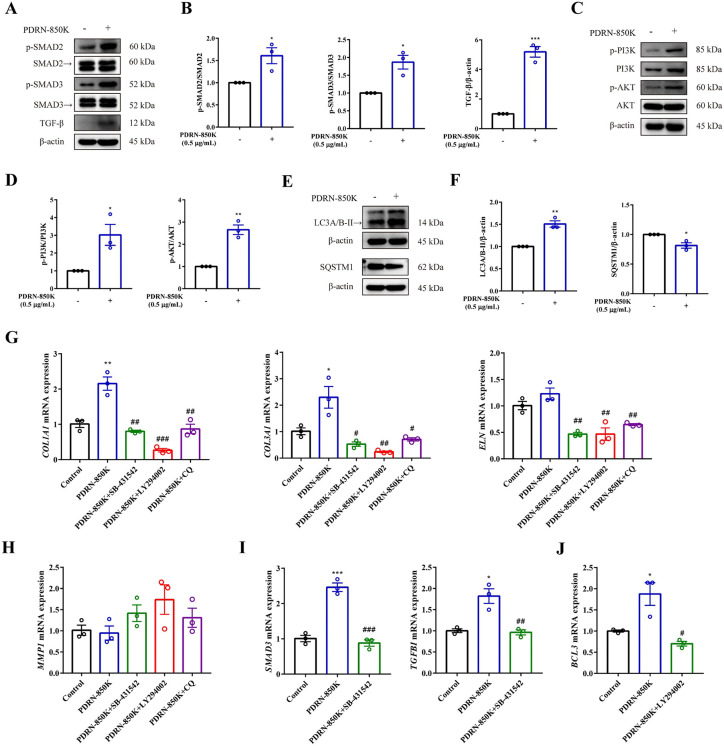
PDRN-850K activates PI3K–Akt, TGF-β/Smad, and autophagy-related signaling in basal human dermal fibroblasts. (A) Western blot images showing phosphorylation of SMAD2 and SMAD3 and expression of TGF-β following PDRN-850K treatment. (B) Quantification of p-SMAD2/SMAD2, p-SMAD3/SMAD3, and TGF-β/β-actin ratios. (C) Western blot images showing phosphorylation of PI3K and AKT. (D) Quantification of p-PI3K/PI3K and p-AKT/AKT ratios. (E) Western blot images of autophagy-related markers LC3A/B and SQSTM1. (F) Quantification of LC3A/B-II/β-actin and SQSTM1/β-actin ratios. (G-H) mRNA expression of *COL1A1*, *COL3A1*, *ELN*, and *MMP1* following PDRN-850K treatment in the presence or absence of pathway inhibitors. (I) mRNA expression of *SMAD3* and *TGFBI* with or without TGF-β pathway inhibition. (J) mRNA expression of *BCL3* with or without PI3K–Akt pathway inhibition. Protein levels were normalized to β-actin. mRNA expression was normalized to control. Data are presented as mean ± SEM (n = 3). *p < 0.05, **p < 0.01, ***p < 0.001 vs. control; #p < 0.05, ##p < 0.01, ###p < 0.001 vs. PDRN-850K group.

Western blot analysis showed that PDRN-850K increased phosphorylation of PI3K and AKT, as well as TGF-β expression and phosphorylation of SMAD2/3, indicating activation of PI3K–Akt and canonical TGF-β/Smad signaling in basal fibroblasts (Fig 1A-1D). In addition, PDRN-850K enhanced autophagy-related signaling, as evidenced by increased LC3A/B-II formation and reduced SQSTM1/p62 levels, consistent with increased autophagic flux (Fig 1E-1F).

To assess functional relevance, ECM gene expression was analyzed in the presence of pathway-specific inhibitors. PDRN-850K significantly upregulated *COL1A1*, *COL3A1*, and *ELN* mRNA levels, whereas inhibition of PI3K–Akt (LY294002), TGF-β signaling (SB-431542), or autophagy (chloroquine) each significantly attenuated these effects (Fig 1G-1J). No significant change was observed in *MMP1* expression, although a general upward trend was noted.

Together, these findings demonstrate that PDRN-850K activates PI3K–Akt, TGF-β/Smad, and autophagy-related signaling in basal human dermal fibroblasts, and that coordinated engagement of these pathways is required for its ECM-promoting activity.

### PDRN-850K shows time-dependent distribution into viable epidermal layers with an endocytosis-sensitive behavior

**Fig 2:**
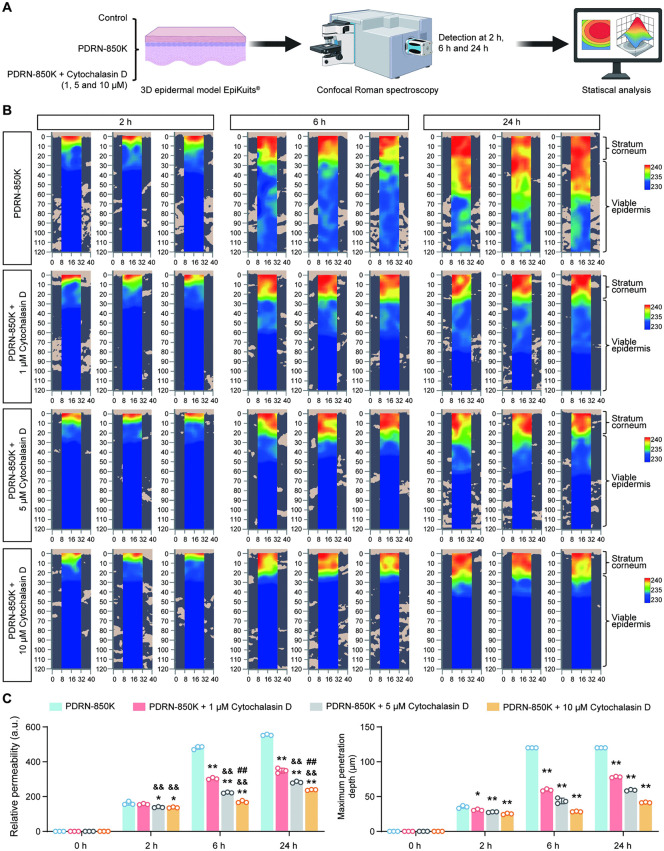
Endocytosis-sensitive skin penetration of PDRN-850K revealed by confocal Raman spectroscopy. (A) Schematic overview of the experimental design for skin penetration analysis. A 3D reconstructed human epidermal model (EpiKutis®) was topically treated with 0.025% PDRN-850K alone or in combination with the endocytosis inhibitor cytochalasin D (1, 5, or 10 μM). PDRN-850K penetration was analyzed by confocal Raman spectroscopy at 0, 2, 6, and 24 h, representing baseline and early, intermediate, and late post-application time points. (B) Raman pseudo-color depth maps showing PDRN-850K distribution in the stratum corneum and viable epidermis at 2, 6, and 24 h. Penetration increased over time and was dose-dependently reduced by cytochalasin D. (C) Quantification of relative permeability (left) and maximum penetration depth (right) of PDRN-850K over time, with or without cytochalasin D. Data are presented as mean ± SEM (n = 3). *p < 0.05, **p < 0.01 vs. PDRN-850K alone; &p < 0.05, &&p < 0.01 vs. 1 μM cytochalasin D; #p < 0.05, ##p < 0.01 vs. 5 μM cytochalasin D.

Skin penetration of PDRN-850K was evaluated using a 3D reconstructed human epidermal model and confocal Raman spectroscopy (Fig 2A), with the nucleic acid–associated Raman peak at 1081 cm^-1^ used for semi-quantitative analysis. Following topical application, PDRN-850K exhibited a time-dependent increase in penetration-associated Raman signal and maximal signal depth at 2, 6, and 24 h (Fig 2B).

**Fig 3:**
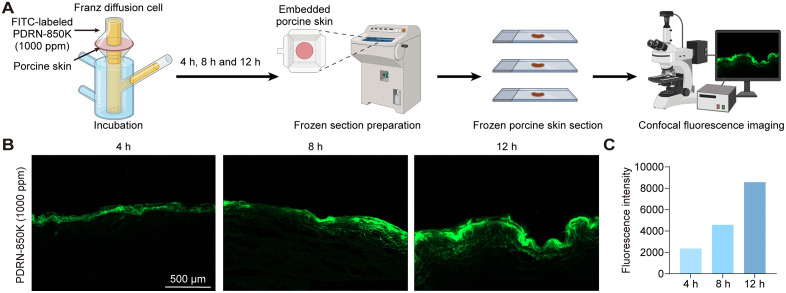
Time-dependent ex vivo skin penetration of FITC-labeled PDRN-850K in porcine skin. (A) Experimental workflow for the Franz diffusion cell assay and confocal fluorescence imaging. (B) Representative frozen sections of porcine skin after topical application of FITC-labeled PDRN-850K solution (1000 ppm) for 4, 8, and 12 h. Fluorescence signal increased over time and extended into deeper skin layers. Scale bar = 500 μm. (C) Fluorescence intensity analysis showing progressive accumulation of FITC-labeled PDRN-850K with increasing incubation time.

**Fig 4:**
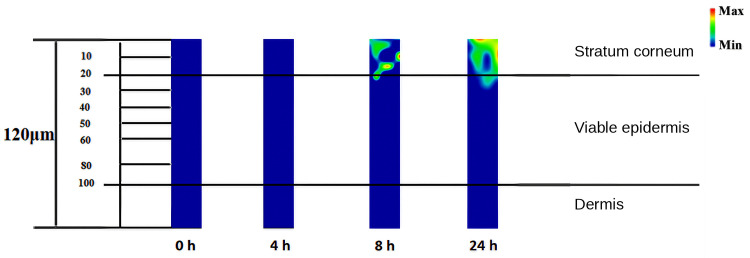
In vivo Raman depth maps showing time-dependent distribution of PDRN-associated signal in human skin after topical application of 0.1% PDRN-850K eye cream. Depth-resolved Raman imaging was performed at 0, 4, 8, and 24 h after application. PDRN-associated signal was minimal at 0 h and 4 h, and became detectable at 8 h and 24 h within the stratum corneum and viable epidermis. Color scale indicates relative signal intensity from minimum (blue) to maximum (red).

Co-treatment with cytochalasin D resulted in a significant, dose-dependent reduction in both penetration signal intensity and penetration depth at all time points (Fig 2C), supporting an endocytosis-sensitive behavior of this process. Because endogenous nucleic acids are present in viable epidermal cells, these Raman findings were interpreted cautiously as supportive evidence for time-dependent distribution of PDRN-associated signal within viable epidermal regions. To further validate these findings, FITC-labeled PDRN was examined in ex vivo porcine skin, where fluorescence imaging showed progressive deepening of signal over time, reaching the dermis at 8–12 h at 0.1% (1000 ppm) as shown in Fig 3. To improve translational relevance, a pilot in vivo human forearm Raman study of the 0.1% PDRN-850K eye cream using the full PDRN-related spectral profile rather than a single 1081 cm^-1^ band showed relative penetration values of 0%, 2.04%, and 3.06% at 4, 8, and 24 h, respectively, with signal distribution beyond 20 μm into the superficial viable epidermis at 8–24 h (Fig 4).

Collectively, these data support time-dependent distribution of PDRN-associated signal into viable skin regions after topical application, although they do not by themselves establish intracellular localization at single-cell resolution.

### PDRN-850K promotes epidermal integrity and broad ECM restoration in UV-damaged ex vivo skin

**Fig 5:**
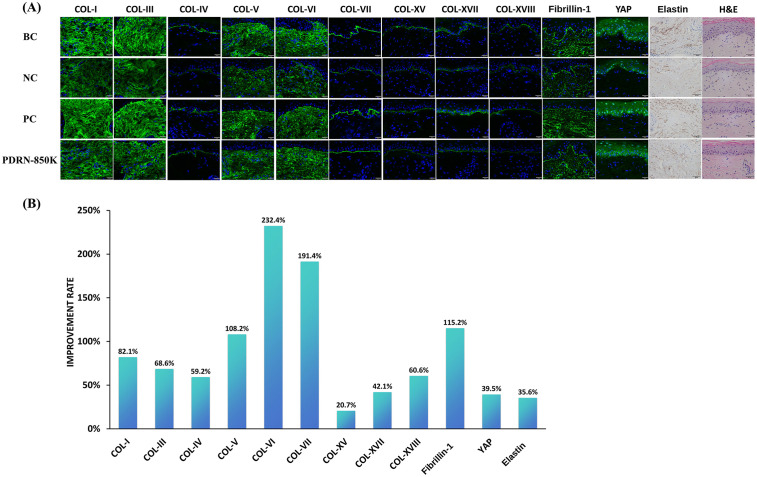
PDRN-850K enhances multiple collagen subtypes, elastic fiber–associated proteins, and YAP expression in UV-damaged ex vivo human skin. (A) Representative immunofluorescence (green) and immunohistochemical staining images showing expression of multiple types of collagens, fibrillin-1, YAP, and elastin in ex vivo human skin. (B) Quantification of protein expression increase levels relative to the UV-damaged control group. Improvement rates are presented as percentage change versus the UV-damaged control. Data are presented as mean ± SEM (n = 3).

In a UV-irradiated human ex vivo skin model, topical application of an eye cream containing 0.1% PDRN-850K significantly increased the thickness of the viable epidermal layer and markedly enhanced expression of multiple ECM components (Fig 5A). Protein levels of collagen I, III, IV, V, VI, VII, XV, XVII, and XVIII, as well as elastin and fibrillin-1, were all significantly elevated compared with UV-damaged controls (Fig 5B). In addition, expression of the mechanotransduction-associated regulator YAP was significantly increased.

Together, these findings indicate that topical PDRN-850K improves tissue morphology and promotes coordinated restoration of diverse collagen networks, elastic fiber–associated proteins, and mechanosensitive signaling in photodamaged skin.

### PDRN-850K eye cream shows approximately two-fold greater clinical improvement than retinol

**Fig 6:**
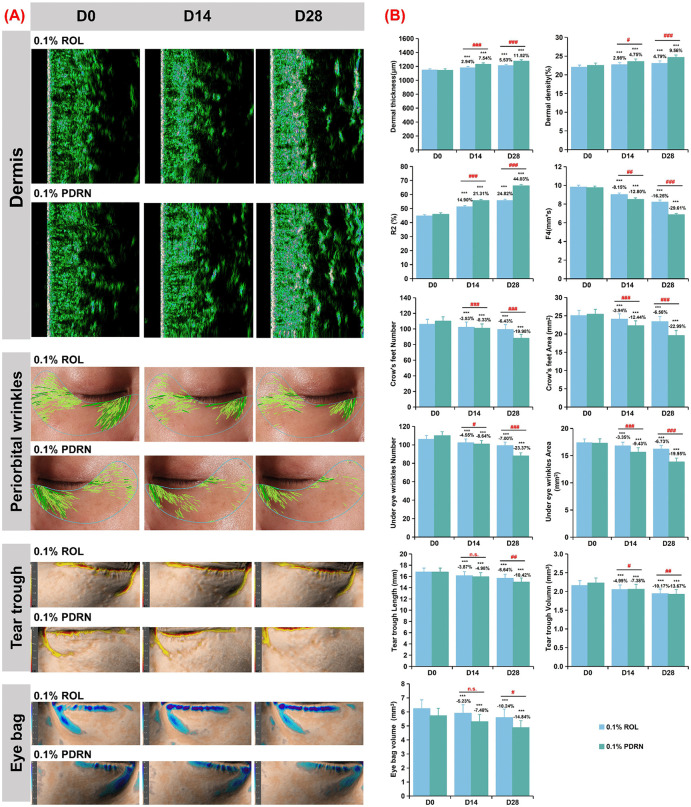
PDRN-850K eye cream demonstrates superior clinical improvement compared with retinol in periocular skin. (A) Representative clinical and instrumental images at baseline (D0), Day 14 (D14), and Day 28 (D28) following twice-daily application of 0.1% retinol (ROL) or 0.1% PDRN-850K (PDRN). (B) Quantitative analysis of dermal structure and periocular parameters. Data are presented as mean±SEM (n = 31). Statistical significance is indicated in the figure.

In a randomized, double-blind, split-face clinical study, topical application of a 0.1% PDRN-850K eye cream produced significantly greater improvements in periocular skin structure than an identically formulated 0.1% retinol eye cream over 28 days. High-frequency ultrasound confirmed that PDRN-850K produced approximately two-fold greater increases in dermal thickness and dermal density compared with retinol. Consistently, three-dimensional imaging demonstrated that reductions in eye bag volume and tear trough depression were approximately two-fold greater on the PDRN-850K–treated side (Fig 6A).

Image-based analysis further showed that PDRN-850K achieved larger reductions in crow’s feet wrinkle area and number at Day 28 (approximately −20% to −23%) compared with retinol (approximately −6% to −7%). Similar superiority was observed for infraorbital wrinkle parameters. Notably, improvements on the PDRN-850K–treated side were evident by Day 14 and exceeded retinol-induced changes measured at Day 28 for multiple wrinkle endpoints (Fig 6B).

Biomechanical assessment demonstrated greater enhancement of skin elasticity (R2) and firmness (F4) with PDRN-850K, with improvements approximately 1.8-fold higher than those observed with retinol (Fig 6B). Both treatments were well tolerated, with no reported adverse reactions.

## Discussion

Chronic UV exposure causes cumulative dermal damage characterized by fibroblast dysfunction, ECM degradation, and impaired tissue repair, ultimately driving the structural features of photoaged skin [18]. Although numerous anti-photoaging strategies have been investigated, relatively few topical agents effectively restore dermal ECM architecture while maintaining tolerability under UV-compromised conditions [19,20]. In this study, we integrate mechanistic, ex vivo, and clinical evidence to elucidate how a defined medium-length PDRN-850K supports dermal ECM restoration following photodamage.

Our previous work demonstrated that topical PDRN-850K promotes ECM recovery in UV-damaged fibroblasts. Building on these findings, the present study clarifies the signaling landscape associated with PDRN-850K–mediated ECM regulation and addresses a key translational gap by directly characterizing its skin penetration behavior. Together, these data advance understanding of PDRN-850K as a topical regenerative strategy for photodamaged skin.

At the cellular level, mechanistic analyses conducted in basal human dermal fibroblasts indicate that PDRN-850K engages PI3K–Akt and TGF-β/Smad signaling alongside modulation of autophagy-related processes, providing insight into its effects on intrinsic fibroblast repair capacity. These studies were conducted in basal fibroblasts to assess direct pathway engagement under controlled conditions and should be viewed as complementary to, rather than substitutes for, our previous transcriptomic observations in UV-damaged fibroblasts. The concurrent activation of these pathways is biologically meaningful, as effective dermal regeneration rarely depends on a single regulatory axis [21,22]. PI3K–Akt supports fibroblast survival and metabolic readiness for anabolism [23], TGF-β/Smad governs transcriptional programs for collagen synthesis and matrix organization [24], and autophagy maintains proteostasis and cellular quality control required for sustained ECM production—functions that are frequently compromised in photoaged fibroblasts [25]. Although *MMP1* showed a modest, non-significant upward trend, this was not indicative of a net catabolic response given the robust induction of collagen- and elastin-related genes, and may instead reflect physiologic matrix turnover during ECM renewal. Consistent with this interpretation, our previous transcriptomic analysis in UV-damaged fibroblasts showed downregulation of *MMP1*/*MMP10* together with activation of pro-repair ECM programs under photodamage-relevant conditions. This coordinated signaling profile contrasts with many classical topical anti-aging ingredients, which are commonly framed around one dominant mechanism, such as retinoid-driven nuclear receptor signaling, antioxidant activity, or metabolic support [26,27]. By simultaneously supporting anabolic signaling, matrix program execution, and intracellular homeostasis, PDRN-850K may promote a more integrated pro-repair fibroblast state, favoring durable ECM restoration rather than transient collagen stimulation. Importantly, interrogating these pathways in basal fibroblasts allowed mechanistic interpretation under controlled conditions without confounding effects of acute phototoxic stress, supporting the view that enhanced intrinsic fibroblast competence can subsequently be mobilized in photodamaged tissue contexts.

A major translational challenge for nucleic acid–based topical agents lies in their ability to traverse the epidermal barrier and reach viable skin layers [28]. Here, confocal Raman spectroscopy in a reconstructed epidermal model demonstrated a time-dependent increase in PDRN-associated signal within viable epidermal regions, and this signal was attenuated by cytochalasin D. The added FITC-labeled ex vivo porcine skin study and pilot in vivo human Raman study provide orthogonal support for topical delivery of exogenous PDRN into viable skin regions and, in the porcine model, into the dermis. Together, these findings support the feasibility of topical PDRN-850K delivery and help explain how this macromolecular DNA preparation may exert biological activity after topical application.

At the tissue level, PDRN-850K treatment enhanced the expression of multiple collagen subtypes, elastic fiber–associated proteins, and the mechanotransduction regulator YAP in UV-irradiated ex vivo skin. Dermal aging involves coordinated deterioration across interconnected collagen networks with distinct structural and organizational roles [29], and selective upregulation of a single collagen species is unlikely to restore functional tissue architecture. In this context, the multi-collagen response associated with PDRN-850K suggests a more comprehensive reconstruction of dermal ECM structure than has been reported for many established topical actives, which are typically linked to limited subsets of matrix proteins. The concurrent upregulation of YAP further supports this integrative repair profile. YAP serves as a central mechanotransduction regulator linking ECM stiffness, cytoskeletal tension, and fibroblast biosynthetic activity, and its decline has been implicated in impaired dermal repair during skin aging [30,31]. Because YAP activity is tightly coupled to cellular energy status, matrix-derived cues, and intracellular homeostasis [32], its induction is consistent with the coordinated upstream engagement of PI3K–Akt, TGF-β/Smad, and autophagy pathways, reinforcing fibroblast–ECM feedback and promoting durable dermal remodeling following photodamage.

The clinical relevance of these findings was supported by a randomized, double-blind, split-face study comparing topical PDRN-850K with retinol at identical concentrations. Under equivalent formulation conditions, PDRN-850K produced greater improvements in periocular dermal structure, wrinkle parameters, and tissue firmness, with favorable tolerability in sensitive skin. Although 28 days is a relatively short timeframe for substantial mature dermal remodeling in vivo, and early ultrasound changes may partly reflect transient hydration or plumping effects, published retinoid studies suggest that clinical wrinkle improvement can appear early, whereas clearer histologic remodeling in photoaged skin is more typically documented over longer treatment periods. A 4-week retinol study showed increased type I collagen production in photoaged forearm skin [33], while classic tretinoin trials in photoaged facial skin generally evaluated histologic change over 24 weeks or longer, with papillary dermal collagen deposition reported after 12 months [34,35]. Nevertheless, nonspecific moisturization alone is unlikely to explain the results because the split-face design controlled for the vehicle base and the same 0.1% PDRN-850K eye cream increased multiple ECM-related proteins in the UV-irradiated ex vivo skin model. Thus, the clinical findings are best interpreted as early improvement signals consistent with both tissue-plumping effects and pro-repair activity. While retinoids remain a cornerstone of photoaging management, their use is often limited by irritation and barrier disruption, particularly in periocular regions [36]. In contrast, the repair-oriented profile of PDRN-850K may offer advantages for long-term use in UV-exposed and sensitive skin.

Several limitations should be acknowledged. Raman spectroscopy alone cannot unequivocally distinguish exogenous PDRN from endogenous nucleic acids or establish intracellular localization at single-cell resolution. Although cytochalasin D reduced the Raman signal, this result is best interpreted as evidence for a living-cell-dependent or endocytosis-sensitive behavior rather than a specific endocytic pathway, because cytochalasin D may affect actin-dependent cellular processes more broadly. Endocytosis also cannot explain the initial passage across the stratum corneum. Thus, the present data are most consistent with a two-step process involving slow trans-stratum-corneum passage followed by uptake-sensitive behavior in viable epidermal regions. The measured hydrodynamic size of the 250 ppm PDRN-850K dispersion was 691.5 nm, but this solution-state parameter does not define the transport mechanism across skin. In addition, because macromolecule delivery is vehicle-dependent, the aqueous reconstructed-epidermis Raman model does not fully represent the 0.1% water-in-silicone eye cream used in the ex vivo and clinical studies; although an in vivo Raman study of the actual formulation was added, direct comparison of vehicle effects was not performed. Further studies using cell-level imaging or other orthogonal tracing approaches will be needed to establish intracellular localization more directly. Pathway activation was assessed in basal rather than UV-damaged fibroblasts, although our previous transcriptomic study in UV-damaged fibroblasts supports the relevance of PI3K–Akt, TGF-β/Smad, and autophagy-related pathways in the photodamage context, direct validation of these signaling events under UV stress will be needed in future studies. Longer-term clinical studies will be needed to define durability and to distinguish early hydration- or plumping-related changes from sustained dermal remodeling.

In conclusion, this study demonstrates that PDRN-850K supports dermal ECM restoration in photodamaged skin through coordinated multi-pathway regulation, enabled by time-dependent topical delivery into viable skin regions. By integrating mechanistic insight with ex vivo and clinical validation, these findings provide a translational framework for nucleic acid–based topical strategies in photodermatology and highlight PDRN-850K as a promising approach for managing photoaging-associated dermal degeneration.
